# Perspectives on frailty screening, management and its implementation among acute care providers in Singapore: a qualitative study

**DOI:** 10.1186/s12877-021-02686-w

**Published:** 2022-01-17

**Authors:** Xiao Liu, Mai Khanh Le, Amber Yew Chen Lim, Emily Jiali Koh, Tu Ngoc Nguyen, Naveed Anjum Malik, Christopher Tsung Chien Lien, Jer En Lee, Lydia Shu Yi Au, James Alvin Yiew Hock Low, Shiou Liang Wee

**Affiliations:** 1 Geriatric Education and Research Institute, Yishun, Singapore; 2 Khoo Teck Puat Hospital, Yishun, Singapore; 3 Changi General Hospital, Simei, Singapore; 4 Woodlands Health Campus, Yishun, Singapore; 5 Ng Teng Fong General Hospital, Jurong East, Singapore; 6 Health and Social Sciences Cluster, Singapore Institute of Technology, Dover, Singapore; 7 Yishun, Singapore

**Keywords:** Frailty Screening, Perspective, Healthcare Providers, Implementation

## Abstract

**Background:**

COVID-19 pandemic has reminded how older adults with frailty are particularly exposed to adverse outcomes. In the acute care setting, consideration of evidence-based practice related to frailty screening and management is needed to improve the care provided to aging populations. It is important to assess for frailty in acute care so as to establish treatment priorities and goals for the individual. Our study explored understanding on frailty and practice of frailty screening among different acute care professionals in Singapore, and identify barriers and facilitators concerning frailty screening and its implementation.

**Methods:**

A qualitative study using focus group discussion among nurses and individual interviews among physicians from four departments (Accident & Emergency, Anesthesia, General Surgery, Orthopedics) in three acute hospitals from the three public health clusters in Singapore. Participants were recruited through purposive sampling of specific clinicians seeing a high proportion of older patients at the hospitals. Thematic analysis of the data was performed using NVIVO 12.0.

**Results:**

Frailty was mainly but inadequately understood as a physical and age-related concept. Screening for frailty in acute care was considered important to identify high risk patients, to implement targeted treatment and care, and to support decision making and prognosis estimation. Specific issues related to screening, management and implementation were identified: cooperation from patient/caregivers, acceptance from healthcare workers/hospital managers, need for dedicated resources, guidelines for follow-up management and consensus on the scope of measurement for different specialties.

**Conclusion:**

Our findings indicated the need for 1) frailty-related education program for patients/care givers and stakeholders 2) inter-professional collaboration to develop integrated approach for screening and management of hospital patients with frailty and 3) hospital-wide consensus to adopt a common frailty screening tool.

**Supplementary Information:**

The online version contains supplementary material available at 10.1186/s12877-021-02686-w.

## Introduction

Frailty, defined as a state of decreased physiological reserve and increased vulnerability to stressor events, is a common problem in older persons [1–3]. A standardized definition was developed by Fried et al. in 2001 [2] and the concept gained increased attention only in the last decade. The prevalence of frailty in community-dwelling older adults ranged from 4%-27% in the Asia–Pacific region [4, 5]. In Singapore, frailty is of concern given the ageing population and negative outcomes associated with it, with a reported prevalence of 5% among community-dwelling older adults [6]. The issue of frailty also only received research and policy attention recently in Singapore [7]. According to the search in PubMed database using word “frailty” and “Singapore” in title or abstract, before 2016, there were, on average, only 1–2 publications on frailty each year from Singapore, but this has increased 5–tenfold in the last few years.

Acute care encompasses a range of clinical health-care functions, including emergency medicine, trauma care, pre-hospital emergency care, acute care surgery, critical care, urgent care and short-term inpatient stabilization [8]. In the acute care settings, frailty was present in 50% to 80% of older patients and was significantly associated with increased adverse outcomes after discharge [9–12]. Hospitalisation is a stressor event that can lead to a further deterioration and loss of independence in older people, especially in those who are frail [13]. There is compelling evidence that frailty is a significant predictor of hospitalisation and adverse outcomes after discharge in older people, such as readmission rates as well as increased risks of disability and mortality [1, 14]. A prospective study implementing frailty screening and frailty pathway intervention among geriatric trauma and emergency general surgery patients in an acute hospital showed improvement of patients’ outcomes including the reduction in length of stay, readmission rate, and loss of independence [15].Studies suggested frailty screening in hospital might benefit the patients by risk stratification and providing individualized and optimized care [16, 17]. Hospitals have been increasingly stretched to cope with the influx of frail older patients and changes are needed to be made to address the shortage of beds in the acute hospitals. This is further exacerbated by the COVID-19 pandemic [18]. By identifying frail patients, we could provide more effective and patient-centred care [16]. There is, thus, a need for routine and systematic assessment of frailty in patients admitted into acute hospitals so as to establish the priorities and goals for the individual to ensure that investigations and treatment accord with what matters to them and their families [19].

Frailty is an emerging concept amongst many healthcare providers, especially in those who are not familiar with geriatric care. A recent scoping review reported a lack of comprehensive policies in implementing frailty screening in the healthcare setting [20]. Most older patients were admitted to general medical wards instead of geriatric wards and received disease-based models of care instead of integrated and patient-centric care emphasising functional ability as an important outcome [7].

According to the Intramural Frailty Science Symposium of the National Institute on Aging (USA), the lack of general consensus on the language used to describe frailty and the varied theories on the pathophysiology of frailty are among the barriers that may discourage clinicians from implementing frailty assessments in clinical practice [21]. To effectively develop and implement a frailty screening program in acute care, the perspectives and understanding of frailty among health care providers who care for and treat older patients should be explored. There have been several studies exploring the perceptions of health professionals about frailty and frailty screening of older patients, but these studies looked mainly at general practitioners; there was limited information on health professionals in the acute care setting [22–24].

This study, therefore, explores the following in the acute care setting: (1) level of knowledge about frailty and frailty screening, (2) identify barriers to frailty screening, and (3) identify facilitators of implementation of frailty management program.

## Methods

This is a qualitative study using thematic analysis. We conducted one-on-one in-depth interviews (IDIs) with physicians and focus group discussions (FGDs) with nurses to understand their perceptions and knowledge of frailty as well as the screening and management of the latter. IDI instead of FGD was used for physicians owing, in part, to the challenges of scheduling a common timing for physicians across departments, whereas nurses engaged in shift work, making it feasible to conduct FGDs for them. This study was approved by the local IRB- Domain Specific Review Board of National Healthcare Group (Reference number: 2018/00551). All methods were performed in accordance with the relevant guidelines and regulations.

### Setting

The study involved clinicians from four specialties- Accident & Emergency (A&E), Anesthesia, General Surgery and Orthopedics. An acute hospital from each of the three public health clusters in Singapore [25] was selected. In consultation with the lead geriatricians of the respective hospitals, physicians and nurses from geriatrics and general medicine were excluded as they were considered to already have extensive knowledge of frailty and had taken on the leadership of frail management in acute hospital among all the specialties in Singapore [7]. These four specialties were selected on the basis that they also managed a significant proportion of prefrail/frail patients in the acute setting. Hence, their perspectives on frailty, frailty screening and management may play an important role in subsequent implementation of hospital frailty care programs.

### Participants and recruitment

Purposive sampling where specific clinicians seeing a high proportion of older persons at hospitals from each from three public regional health clusters were selected. Site PI’s were senior geriatricians from each hospital who led the recruitment effort by identifying potential participants who were involved in the clinical management of frail patients. Physicians and nurses received invitation emails with the study details before making their decision to participate. Varied disciplines or specialties and ranks of staff ensured heterogeneity, which, in turn, improved the breadth and depth of the discussion. Focus groups from same hospitals, on the other hand, ensured standardization and also facilitated discussions [26].

### Data collection

Based on our research aims and questions, we developed a semi-structured interview guide. A review of the literature helped to formulate the domains for the guide, which explored frailty awareness and the possibility of implementing frailty screening in acute care. The data were collected between September 2018 and November 2019 from a total of 70 participants (51 IDIs with physicians and three FGDs with 19 nurses). All participants gave informed consent prior to the interviews, which lasted between 20 and 54 min. Participants were first asked about their perspectives on frailty and frailty screening, after which, they were introduced to and asked to comment on six common frailty assessment tools: 1) Fried’s Frailty Phenotype [27]; 2) Frailty Index [28]; 3) FRAIL scale [29]; 4) Clinical Frailty Scale (CFS) [28]; 5) Edmonton Frail Scale [30] and 6) Tilburg Frailty Indicator [31]. Participants were asked about their understanding of frailty, perceptions on the role of frailty in healthcare, frailty screening, and frailty screening tools in relation to their specialty. They were also asked about their practice when handling frail patients, the inhibitors and facilitators of adoption of frailty screening and their preferred frailty screening tool. The interview guide was in Additional file 1. The interviews were conducted by three investigators who were unacquainted with the participants. Field notes were taken during the sessions. The interviews were audio-recorded, transcribed, and thematically analyzed. Thematic saturation was monitored throughout recruitment. After three FGDs and 41 IDIs, there were no new theme generated from the interviews. We conducted another 10 IDIs to confirm and ensure the saturation.

### Analysis

Data from all 51 physicians and 19 nurses were useful and included in the analysis. Thematic analysis [32] was conducted using the framework approach (FA) [33] to incorporate and categorize complex data from both focus group and individual interviews. The development of a preliminary coding framework was based on prior literature review. Each transcript was coded by two out of the four investigators using NVivo 12.0 Pro (QSR International 2020). The investigators coded independently, met afterwards to check and discuss any discrepancies and refine the framework. Final themes derived from the codes were decided by two members most experienced in qualitative analyses.

## Results

Table 1 shows the demographic information and job roles of the participants. Findings were categorized into three major themes: 1) knowledge about frailty, 2) perceived importance of frailty and frailty screening and 3) the barriers and facilitators to frailty screening, frailty management and implementation of frailty screening.

**Table 1:** Demographics and job experience information

	Physician (N = 51)	Nurse (N = 19)
**Age** (mean, SD)	44.9 (9.0)	42.7 (10.7)
**Years of experience** (mean, SD)	18.6 (9.3)	19.8 (11.5)
**Job title (Physician)** (n, %)		
Consultant	16 (31.4)	
Senior consultant	20 (39.2)	
Others	15 (29.4)	
**Job title (Nurse)** (n, %)		
(Assistant) Nurse Clinician and Nurse Manager		12 (63.2)
Others		7 (36.8)
**Specialty** (n, %)		
Accident & Emergency	12 (23.5)	5 (26.3)
General Surgery	14 (27.5)	5 (26.3)
Orthopedics	12 (23.5)	4 (21.1)
Anesthesia	13 (25.5)	5 (26.3)

Table 2 illustrated the themes and sub-themes of our results. The quotes examples were in the Additional file 2.

**Table 2:** Themes and sub-themes

Theme	Sub-theme
Knowledge about frailty	The knowledge levels about frailty were inconsistent
	Frailty information was mainly obtained from work-related activities
	Frailty is characterized as loss of physiologic reserves
	Frailty is generally but not necessarily age-related
	Frailty dimensions includes not only physical, but also cognitive and psychosocial conditions
Perceived importance of frailty and frailty screening	Frailty is important in the context of the increasing aging population and proportion of older patients in hospital
	Frailty screening helps to identify those patients at higher risk of adverse clinical outcomes
	Patients with frailty requires modified treatment and/or more intensive clinical care to achieve better outcomes
	Frailty screening provides information for decision making and prognosis estimation
Barriers and facilitators to frailty screening, frailty management and implementation of frailty screening	Cooperation from patient/caregivers
	Acceptance from healthcare workers/hospital managers
	Dedicated resources
	Guidelines for frailty management
	Uniform scope of measurement among specialties

### Knowledge about frailty

The knowledge level of frailty among participants distributed variously. One third of the participants showed extensive understanding of frailty by clearly explaining the definition of frailty, related clinical conditions and risks as well as accurately identifying some of the screening tools. In contrast, the other two third of the participants admitted that their knowledge related to frailty was inadequate. Among the latter group, some referred frailty as a relatively novel concept to them that was not taught at the undergraduate, postgraduate and advanced specialist training levels. Instead, participants often learnt about the concept during work-related activities such as conferences.

Frailty was mostly understood as a physical and age-related construct. Most participants defined frailty as (1) a decline in functionality where patients lose their mobility and ability to perform activities of daily living (ADLs), (2) weakness in strength and appearance, (3) physiological deterioration (e.g. deterioration in bone, muscle mass, organs), (4) increase in health risks, and (5) having comorbidities. Frailty was generally thought to be age-related. However, some participants acknowledged that frailty was a result of medical problems, hence, it could present in younger patients too.

Although less mentioned, a number of participants did recognize that frailty could also encompass social, psychological, and cognitive dimensions, besides physical conditions. Patients with cognitive impairment, psychiatric issues, or low social economic status were relatively more vulnerable, thus these dimensions should also be considered in defining frailty.

### Perceived importance of frailty and frailty screening

Despite varying levels of knowledge about frailty in acute hospital, 52 out of 70 participants (38 doctors and 14 nurses) recognized the importance of frailty, the reasons for which can be summarized into four parts:

First, nearly half of the participants stated frailty was of importance in the context of the aging population expansion. Some of them mentioned the statistics that they had awareness of the increasing population of older adults in Singapore and some also witnessed the number of older population occupied a rapid enlarging proportion of patients in hospitals, which made frailty a critical issue for not only the geriatrics but also other specialties as well.

Second, one third of the participants suggested frailty screening was beneficial to identify those patients at higher risk of adverse clinical outcomes. They mentioned there were several risks related to frailty including but not limited to poorer clinical outcomes and higher risk of complications compared to patients who were not frail. Hence, frailty screening is an opportunity to identify vulnerable patients early.

Third, more than half of the participants agreed that specific clinical care or treatment were necessary for patients with frailty to achieve better outcomes. Doctors from different specialties provided various comments on the modifications over the normal routine. The most common opinion from general surgery and orthopedic surgery was to give patients with frailty pre- and post-operation rehabilitation to strengthen their muscle, to reduce the risk of operation complications, and to enhance post-operation recovery. Some anesthesia pointed out they would adjust the dose of drugs so as to prevent postoperative cognitive dysfunction and also be cautious about patients’ normothermia during operation. Nurses from all the included departments reached the consensus on the demands for providing extra attention for patients with frailty especially on fall prevention.

Last, almost half of the participants stated that frailty screening provides information for decision making and prognosis estimation. Were the screening results taken as the baseline reference, doctors could better diagnose the disease and prognosticate how patients would respond to treatment. The screening results were useful to provide individualized care to patients with frailty because they could also be used in the communication between doctors and patients’ families to set more attainable care goals, choose care in the patient’s best interest, decide the treatment plan. Some doctors also mentioned frailty was one of the considerations when they decide the disposition and discharge plan.

### The barriers and facilitators to frailty screening, frailty management and implementation of frailty screening

#### Cooperation from patient/caregivers

A number of participants mentioned that the lack of cooperation from patients or caregivers, for both subjective and objective reasons, could impede the implementation of frailty screening. For patients went to hospital for a certain disease (not for frailty) or due to emergency, frailty screening seemed to be an unnecessary exam for them because they aimed their diseases to be treated, especially among those who had no or limited knowledge about frailty and frailty screening. Some patients might feel uncomfortable answering questions related to their own social status (household income, living status, etc.) due to privacy concerns, or might not be forbearing enough to answer questions that do not appear relevant to their emergent health issues.

At the same time, difficulty in communication was a major reason for inaccuracies in patients’ answers. For example, some patients might lack the capability to answer independently (e.g. loss of mental capacity, physically unable to communicate, inability to read or respond, language barriers, etc.), and may require assistance from their family members or other caregivers to communicate the answers. Hence, the fidelity of the answers could be compromised since the caregivers may not comprehend or understand the patient’s answers, e.g. foreign domestic helpers who are less conversant in local languages and/or dialects. In addition, comprehensive physical assessments required of frailty assessments would impose additional burden on busy clinicians, even for the less frail patients, since the former takes extra time, effort and resources (manpower, funds, etc.) to carry out. Frailty diagnosis and treatment are not covered by any insurance plans, hence, they would be hesitant to follow the instructions.

#### Acceptance from healthcare workers/hospital managers

Although a number of the participants acknowledged the importance of frailty and frailty screening in the acute hospital, 18 out of 70 participants felt that frailty screening was not important to their specialty. Frailty screening was thought to be relevant to only a certain group of patients, such as older patients, and, even then, only certain aspects of frailty were relevant to their specialty, which would have been assessed anyway with their existing practice.

A few participants felt that frailty screening would not impact their medical decision. Most of them were A&E physicians who consistently expressed that their main responsibility was to respond to patients’ acute needs such as life-threatening symptoms, which did not call for frailty screening. Other participants also felt that frailty screening was unimportant as they thought that their colleagues and hospital management felt the same way. Not all healthcare workers would be willing to conduct frailty screening, due to their limited knowledge of frailty screening tools, perceived complexity of conducting frailty assessment. In addition, some participants especially the nurses mentioned they would not like this additional burden of screening to their workload, even though they know the importance. Although some tools, such as the CFS, were proven to be easy to administer, there still appears to be some resistance from healthcare providers*.*

#### Dedicated resources (manpower-workload, funding, space)

More than half of the participants raised concerns related to the limited resources available to perform frailty screening, especially among participants from A&E. They felt that the current workforce was already overwhelmed with existing duties and complex clinical tasks. However, some nurses felt that frailty screening should be conducted by the physicians while physicians thought otherwise. Nevertheless, the analysis showed that majority of the nurses and physicians found it appropriate to have geriatricians perform the screening instead. In addition, insufficient funding, lack of tools and equipment, and inadequate space were also raised as barriers to effective frailty screening. An example would be gait speed measurement which requires extra space to be carried out. Although some participants admitted the importance of frailty screening, others, particularly from A&E, shared that the availability of limited resources restricted them to “eyeball” screening, instead of a systematic screening for frailty. In summary, the benefits from frailty screening could also be too minimal and would be offset by the logistic and resources challenges that come with added screening.

#### Guidelines for frailty management

An overarching concern by participants was the lack of follow-up care after frailty screening. Without proper follow-up, clinical management guideline or discharge plans, frailty screening could be regarded as futile and a waste of effort and resources. It was also one of the reasons why some healthcare professionals had lacking acceptance on frailty screening. Some participants suggested that the specific frailty management guideline could be helpful to let them know how to make targeted and better practice among patients with frailty, so that they would get the meaningful value of frailty screening.

#### Uniform scope of measurement among specialties

Different specialties often approach the assessment, care and management of patients from very diverse angles. For example, while all were focused on the patient’s primary conditions, Anesthetists tended to be less concerned about a comprehensive medical history and with activities of daily living. As a result, the different specialties had varied opinions about frailty screening tools. When asked to comment on the six frailty screening tools presented, participants shared their thoughts on the advantages or disadvantages of each tool. Participants felt that the Fried’s Frailty Phenotype was quick to administer while the Frailty Index was comprehensive. They found that the Edmonton Frail Scale and Tilburg Frailty Indicator were simple to administer. However, participants felt that some of these tools were not suitable for use in their departments. For example, participants from A&E were not in favor of using any of the tools even though they thought CFS was the only one relevant to their work; while participants from surgery favored using the tools and specifically mentioned Fried’s Frailty phenotype, Frailty Index and CFS as being relevant to their daily jobs.

Perspective of the screening tools showed clustered effect by specialty as well as some random effects by individual, which resulted in conflicting pros and cons for the same tool. For example, some A&E physicians claimed Fried’s Frailty Phenotype was difficult to administer and time consuming because they did not have muscle strength and gait speed test as normal practice and their time was limited, while orthopedic physicians thought it simple and quick to do.

Table 3 showed detailed information of all the mentioned advantages and disadvantages.

**Table 3:** Perspectives of 6 proposed tools from stakeholders

Name of the Tool	Pros	Cons
Fried’s Frailty Phenotype	Objective (N = 40) Relevant to work (N = 12) Quick to administer (N = 11) Simple to do (N = 8)	Not comprehensive (N = 21) Takes time (N = 11) Difficult to administer (N = 11) Not suitable for department (N = 9) Not useful (N = 5)
Frailty Index	Comprehensive (N = 46) Relevant to work (N = 19) Straightforward (N = 9) Simple to do (N = 8) Objective (N = 8)	Takes time (N = 43) Difficult to administer (N = 25) Not suitable for department (N = 14)
FRAIL Questionnaire	Quick to administer (N = 38) Simple to do (N = 37) Self-administered (N = 24) Relevant to work (N = 9)	Not comprehensive (N = 27) Not suitable for department (N = 15) Self-administered (N = 14) Not Useful/Not meaningful (N = 13) Difficult to administer (N = 7)
Clinical Frailty Scale	Quick to administer (N = 50) Have pictures (N = 23) Simple to do (N = 23) Relevant to work (N = 19) Objective (N = 7)	Subjective (N = 21) Not Comprehensive (N = 10) Not suitable for department (N = 9)
Edmonton Frail Scale	Relevant to work (N = 25) Comprehensive (N = 21) Quick to administer (N = 20) Simple to do (N = 14) Simple scoring (N = 8)	Takes time (N = 18) Difficult to administer (N = 15) Not suitable for department (N = 13)
Tilburg Frailty Indicator	Comprehensive (N = 17) Self-administered (N = 12) Simple to do (N = 10) Relevant for work (N = 6)	Not useful (N = 18) Difficult to administer (N = 14) Not applicable to Singapore (N = 10) Self-administered (N = 8) Takes time (N = 9)

*N* number of participants mentioned the point

## Discussion

This study sought to explore healthcare professionals’ understanding of frailty, frailty screening practice and perceived barriers to frailty screening and management in the acute care setting. Despite varied levels of understanding about frailty, majority of the participants (n = 52) recognized the importance of frailty and frailty screening. However, challenges to the implementation of frailty screening were expressed by participants. Implementation of frailty screening should be informed by proper evaluation of the feasibility and applicability of the tools in a structured and standardized manner. The expressed barriers should be addressed by closing the knowledge gap of the clinicians. It is clear that hospital managers and clinicians need to work together to adopt clear frailty management plan and workflow to go with frailty screening. In addition, patients and caregivers should be educated on the importance of frailty as a determinant of their care outcome.

### Comparison with existing literature

Stakeholders’ level of understanding on frailty seen in our study was in line with results from previous studies in Canada [22, 34], especially from the perspective of aging as well as physical, cognitive and social factors. Similar to our findings, these two studies, conducted on participants from health clinics, found that healthcare providers possessed various levels of understanding regarding frailty, and recognized aging and physical factors to be the most important aspects of frailty. In our results, more than two thirds of the participants agreed frailty and frailty screening in hospital was of importance. The results of the meeting between six major international, European and US societies along with 7 experts in the area of frailty reached the same conclusions.

Previous literature and guidelines in primary care settings found that frailty screening was often thought to help in the reduction of complications and mortality of patients [35–38]. It is worth noting, however, that most of the patients in primary care were in the prefrail stage whereas those in the acute care settings were often frail. Hence, while frailty screening was carried out in primary settings to avert the frail status of patients, this may not be practical or applicable in the acute settings. Another study with surgeons showed that frailty screening could lead to less adverse outcomes in the peri-operative period [39, 40].

Barriers identified such as the lack of resources (time, manpower) and guidelines in the healthcare system were consistent with an integrative review conducted in 2019 [41]. However, our study recognized an additional barrier, where a minority of acute care clinicians still did not perceive that frailty screening and, therefore management as important. The need to better define frailty and the use of a standardized tool to ensure consistent and transferable results as suggested by the participants was also shown by several studies [34, 38]. A study in the Netherlands [42] found that the lack of guidelines for care after frailty screening prevented frailty screening programs from being implemented. In the A&E setting specifically, due to patients’ reduced function and increased vulnerability, it would be challenging to obtain accurate frailty screening results [34]. In addition, an integrative review of 14 screening tools in acute care settings suggested that overwhelming workloads, uncooperativeness among members of the multidisciplinary team and insufficient follow-up management support could hamper the implementation of frailty screening [41]. Similar to our findings, interdisciplinary teamwork between professionals, specialties and departments was suggested as a solution to this issue [42].

### Implementation of frailty screening in acute settings

Our findings on stakeholders’ understanding or perspectives on frailty, its role in hospital care, screening and management provide valuable information for the implementation of frailty screening and management programmes in the acute care setting in Singapore. Effective implementation of changes in the healthcare system or hospital through initiatives such as advance care planning will require cultural and behavioural transformation [43]. Some of the possible measures to adopt in the implementation process include:

#### Education program for patients/care givers and stakeholders

The benefits of frailty screening in acute hospital have been well-recognized in the literatures. However, we found disparate perceptions on the importance of frailty screening among our participants, which is consistent with the results of another study on the understanding of frailty identification and management in orthopaedic practices [40]. This unveiled the barrier on the acceptance of frailty screening which highlighted the needs of education to achieve better understanding of frailty among health professionals. Designing and customizing education programs for both patients/caregivers and stakeholders are essential to improve the probability of getting buy-in. Patients/caregivers would be more likely to cooperate if they were aware and understood more about the importance of frailty and frailty screening, and how much frailty screening would benefit them. Possible solutions might include disseminating easy-to-read flyers to the community centres or public educational campaigns. Meanwhile, health professional education programs on frailty, highlighting the importance of frailty screening, and targeted frailty screening methods for physicians, nurses and administrators should be implemented in a formal setting as well as complementary training to enhance the competency and confidence of stakeholders when handling frail patients and dealing with frailty issues. Such training programs could also help healthcare providers appreciate the cost-effectiveness of frailty screening [44] and garner more buy-in from the higher level stakeholders. Nurses in one hospital mentioned that attending relevant education programs on frailty screening could promote more awareness of frailty and frailty screening among nurses. Such education programs should form part of the strategy towards implementing and sustaining hospital wide frailty screening and management programs.

#### Hospital wide and inter-professional involvement

As indicated from our results, shortage of dedicated resources was a barrier to the implementation of frailty screening in acute hospital. The inter-professional collaboration would be helpful to fill in the knowledge and expertise gaps, especially among non-geriatric specialties. Meanwhile, a hospital wide workflow of the screening and management of patients with frailty would facilitate more efficient utility of the manpower and other resources in hospital. As previous literature suggested, early Comprehensive Geriatric Assessment involving a multidisciplinary team [37] will improve collaboration, communication and the sharing of information with minimal omissions and duplication, which would help conserve resources from the outset. Health providers from the various departments would benefit from geriatric expertise and be able to utilize results from frailty screening to make their own informed medical decisions. Last but not the least, inter-professional collaboration would allow frailty screening results to be obtained and delivered across specialties quickly and effectively.

#### Adopting a common frailty screening tool

In our study, there were inconsistent perspectives on frailty screening tools clustered by the different clinical experience and knowledge background among participants, which presenting a barrier to reaching the consent on routine frailty screening in hospital. In order to ensure that there is a common understanding of frailty and a seamless flow of information between specialties, a common frailty screening tool that takes into account the different needs of the various specialties isa imperative. The Clinical Frailty Score (CFS) [28] and Edmonton Frail Scale [30] were the most favored tools among the participants. According to a scoping review of 204 articles, CFS seemed to be the most commonly used tool among 13 established tools, followed jointly by the Frailty Index and the Frailty Phenotype [16]. It is therefore possible that the standardized tool can be adapted from the CFS and/or Edmonton Frail Scale. It would also be necessary to involve the multidisciplinary or interprofessional teams to adopt a standardized frailty tool, taking into account not only the needs, but also the challenges of administering the tool, in terms of manpower and resources constraints, that each department faces. Finally, we found that most participants of this study were not aware of currently available frailty screening tools. Hence, should a standardized frailty screening tool be adopted, knowledge translation approaches should be applied to scale up and sustain its use in the healthcare system. This would include strategies to build and develop awareness, training and quality improvement programs, which would ultimately lead to improved quality of care of patients.

### Strength and limitations

To our knowledge, this study is among the first to explore the acute care providers’ perception and attitudes towards frailty and frailty screening. Our findings could inform healthcare administrators and policy makers on potential knowledge and service gaps associated with frailty management and improve acute care of frail patients. In addition, by collating the views of non-geriatric physicians and nurses, the study was able to unveil the general perspectives of those who might not be familiar with frailty issues, despite caring for a large number of frail patients.

Due to the convenience recruitment approach, we expect certain level of selection bias to be present. However, care was put into recruiting healthcare providers of various age, gender, seniority, and level of experience to limit the effect of bias as much as possible.

## Conclusion

In general, nurses and physicians in acute hospitals in Singapore share similar perspectives on the importance of frailty and frailty screening although their understanding of the latter is varied. This study revealed important barriers to frailty screening in the acute care setting, which informed our recommendations on how to implement frailty awareness and screening in the acute care setting in Singapore. Interprofessional collaboration, a universal screening tool and education efforts to close the knowledge and expertise gaps in frailty care are necessary ways towards successful implementation of frailty screening and management in the acute care setting.

## Supplementary Information

**Additional file 1.** Interview guide.

**Additional file 2.** Themes and quotes examples.

## Data Availability

The datasets used and/or analyzed during the current study are available from the corresponding author on reasonable request.

## Abbreviations

IDI: In Depth Interviews
FGD: Focus Group Discussions
A&E: Accident & Emergency
CFS: Clinical Frailty Scale
FA: Framework Approach
ADLs: Activities of Daily Living
