# Supplementary material for: Perspectives on frailty screening, management and its implementation among acute care providers in Singapore: a qualitative study
Source: BMC Geriatr. 2022 Jan 17;22:58. doi: 10.1186/s12877-021-02686-w (PMC8762449; doi:10.1186/s12877-021-02686-w)
Supplement: Supplementary file 1 — Additional file 1. Interview guide. [file 12877_2021_2686_MOESM1_ESM.docx]

**Additional file 1: Interview guide**

**Qualitative study of stakeholder understandings and experiences**

**of frailty and frailty screening in acute care settings in Singapore**

Semi-Structured Interview Guide

Note: The questions here only serves as a guideline to help obtain information relevant to the study. The questions do not need to be asked in order and need not be asked again once the relevant information is obtained.

**INTRODUCTION AND CONSENT PROCESS** [5-8 MINUTES]

Hello, I am [Name]. [Introduce any other research team members, if any.] Thank you [Interviewee’s Name] for agreeing to this interview today. This study will greatly benefit from your sharing and inputs. I/ We would love to learn more about your experiences with the frail and understand your opinions on frailty, frailty screening and common frailty screening tools.

This discussion will be approximately 60 minutes. I/ We will be audio recording this discussion in order to capture the thoughts and ideas generated from this session. We will place the audiotape [here/ other location]. Should you feel uncomfortable to answer any questions in this interview, you may refuse to answer. You may also withdraw from this study at any point in time of this research.

I/ We understand the importance of privacy and confidentiality. I/ We reassure you that the information that is given to us in this discussion is completely confidential. Your identity will be kept anonymous through the pseudonym that will be assigned to you.

Before we begin, do you have any questions regarding the research project, or this discussion? [Answer questions if any. If there are no questions, please proceed.] Should you have any questions after you have completed the study, you can always contact the principal investigator of this study. His contact details are in the consent form that you will receive.

[Consent form for the participant to sign before proceeding]

All right, we will start the interview now.

[Turn on audio recording device now]

1. **ICEBREAKER** [5 MINUTES]

**Q1** Could you tell us a little more about yourself and what you do in your job?

1. How long have you been working in your current role?
2. What kinds of patients do you work with?

Could you describe the type- age, physical profile, sources and other characteristics of the patients? Refer from ED or other department?

1. **EXPERIENCE AND PERSPECTIVES ON FRAILTY** [15 MINUTES]

**Q2** What does the word- ‘frailty’ mean to you?

1. What other aspects of ‘frailty’ are there (besides the physical aspect)?
2. Based on your experience, how relevant are these other ‘frailty’ aspects to your clinical work?
3. What are the trends you encounter with your frail patients?

**Q3 How does frailty influence your clinical work?**

Based on your experiences, please describe its importance to clinical outcomes.

**Q4** Could you describe your experiences with frail patients at the [specific work location(s) e.g. emergency departments/ wards/ outpatient clinics etc.]?

1. How would you identify if a patient is frail?
2. What are the considerations you take when you encounter frail patients?
3. How differently will you treat your frail patients? Management plan?
4. **KNOWLEDGE OF FRAILTY SCREENING TOOLS** [20 MINUTES]

**Q5** What are the screening tools that you currently use to identify frailty?

1. What are the benefits of carrying out your current frailty screening tools?
2. What are the challenges of carrying out your current screening tool?

**Tools will be printed out and shown to the participants:**

- Fried’s frailty phenotype criteria
- The Frailty Index
- FRAIL scale
- The Clinical Frailty Scale
- The Reported Edmonton Frail Scale
- The Tilburg Frailty Indicator

**Q6** Which of these tools are you most familiar with?

**Q7** Briefly, what do you think are the pros and cons of these tools in the context of your work?

1. **ATTITUDES TOWARDS FRAILTY SCREENING** [15 MINUTES]

**Q8** In your opinion, how necessary is frailty screening?

**Q9** If you had your way, how do you think frailty screening should be carried out?

1. Who do you think should carry out the frailty screening?
2. Where is the best place to carry out the frailty screening?
3. How long should the frailty screening be?
4. In the context of the patient’s journey, when should frailty screening be done?

**[[Q5** What do you think about incorporating a frailty screening routine in your clinical work?

1. What will be an ideal frailty screening routine in the setting of your clinical work?
2. How do you intend to incorporate the frailty screening routine in the setting of your clinical work?
3. Who do you think should carry out frailty screening in the setting of your clinical work?

- **NOTE:** The difference between this and Q9 is that Q5 only asks about incorporating the frailty screening routine in the clinical setting of the participant. ]]

**Q10** If you had to choose these tools for frailty screening based on how you think frailty screening should be carried out, which of these tools would you choose to use for frailty screening?

1. What is the reason for this choice?
2. Or if the reason seemed clear in Q6, clarify if that is the reason.

**Q11** Any final comments with regards to frailty, frailty screening and frailty screening tools?

All right, we have come to the end of the interview. Thank you so much for sharing your views and perspectives on frailty and frailty screening with us today! We truly appreciate your time, and the study will benefit greatly from all these inputs. If you have any further questions, please feel free to email us.
