# Supplementary material for: Perspectives on frailty screening, management and its implementation among acute care providers in Singapore: a qualitative study
Source: BMC Geriatr. 2022 Jan 17;22:58. doi: 10.1186/s12877-021-02686-w (PMC8762449; doi:10.1186/s12877-021-02686-w)
Supplement: Supplementary file 2 — Additional file 2. Themes and quotes examples. [file 12877_2021_2686_MOESM2_ESM.docx]

**Additional file 2: Themes and quotes examples**

| Theme | Sub-theme | Quotes example |
| --- | --- | --- |
| Knowledge on frailty | The knowledge levels on frailty were inconsistent. | *“Frailty means that there’s progressive decline of the patient’s function and that puts him in a vulnerable state. Our geriatric nurse use CFS. If they’re between the score four to six then we consider pre-frail, if higher than that, then frail.”* [F006, A&E doctor]  *“Yeah, sad to say because I think it’s something that’s not really in the knowledge base of most orthopedic surgeons, if not all. And perhaps also relatively it’s something that is new. Because when I was a student, we never actually had such thing, that’s for sure.”* [F063, Orthopedic doctor]  *“I have a feeling that you may not be getting what you need from me because honestly, I really don’t know about this.”* [F038, A&E nurse] |
|  | Frailty information was mainly obtained from work-related activities. | *“It’s quite new concept for us. I never had like a proper teaching on frailty, to be honest.”* [F065, Orthopedic doctor]  *“My medical school was 10 years ago, so the emphasis wasn’t that strong on geriatrics at that time, I don’t think frailty was in the curriculum.”* [F022, General surgery doctor]  *“Okay, we hear it being mentioned once in a while by our geriatricians, they also talk about it in the notes”* [F036, Anesthesia nurse]  *“I have only heard about it in the last 2 years I think when I attended some conferences for anesthesia of course.”* [F008, Anesthesia doctor] |
|  | Frailty is characterized as loss of physiologic reserves. | *“Frailty means the patient of the age group of those who have weakened over a period of time with regards to their muscle mass, their bone density, and the overall immunity of the patient. So their physiological reserve is diminished significantly compared to other people of the same age group.”* [F042, Anesthesia doctor]  *“Frailty is a spectrum or condition where you actually assess how weak a person is, in terms of his ability to withstand stress and his ability to cope with some added insight or illness which come to him, and whether that will cause a deterioration in his activities of daily living or his outcomes if an intervention is done.”* [F040, General surgery doctor]  *“Frailty what I understand is like, patient who lost their muscle, they lost their weight, then feel lethargy, feeling very weak, cannot undergo their day-to-day activities because of the– progressing in the age.”* [F067, A&E doctor] |
|  | Frailty is generally but not necessarily age-related. | *“It does not really mean to go according to patient’s chronological age. So you can have patients who are young but yet frail. Or patients who are old, very old but yet they’re not frail.”* [F046, Orthopedic nurse]  *“Honestly, okay, you will see association between age and frailty – the older a patient gets, the more likely the patient going to be frail. I think frailty is the nature; is a part of ageing process.”* [F005, General surgery doctor]  *“I suppose you could be young and if you have a certain medical condition, you could be frail as well, and then the other converse is also not necessarily true, you can be elderly and still very strong.”* [F001, A&E doctor] |
|  | Frailty dimensions includes not only physical, but also cognitive, and psychosocial conditions. | *“Yeah, so if a patient is dementia, to me that's one form of frailty as well that we have to take note of.”* [F021, Anesthesia doctor]  *“in terms of mental frailty there’s a deterioration in their cognition or memory. So there’s two aspects, the mental and physical aspects to see whether they qualify.”* [F040, General surgery doctor]  *“A patient who is of a lower social-economic status will probably have less access to medicine, probably less access to resources and therefore might be considered frail.”* [F041, Anesthesia doctor] |
| Perceived importance of frailty and frailty screening | Frailty is important in the context of the increasing aging population and older patients in hospital. | *“Ideally, all doctors should be able to do a good frailty score, because as I’ve said, one-third of Singapore’s population is going to be above sixty-five in 2050 which is not very far from now.”* [F002, Anesthesia doctor]  *“Well regarding my ward, which is orthopedic surgery ward, we are seeing more and more geriatric age group patients. So definitely the frailty screening is very important.”* [F053, Orthopedic nurse]  *“Because I see a significant proportion of elderly patients, even if I am not doing the sub-specialty called Geriatric Emergency Medicine, I do need to know a bit about frailty, we have to, because the trend is that we are seeing more and more elderly patients. And you know, the fact that the aging population is going to double in the next 10 years.”* [F001, A&E doctor] |
|  | Frailty screening helps to identify those patients at higher risk of adverse clinical outcomes. | *“Frailty screening allow us to identify the high-risk cases. If a patient is frail, it means they physically weak, then the surgery become a challenge to them. So you worry about the post-operative complications which affect the patient outcome very much. So patient with this condition usually requires to be screened and requires some assessment before we proceed with operation.”* [F068, A&E doctor]  *“I have said many times to my department that we should do frailty scoring. This is important because we know frailty scoring is associated with length of the stay, the mobility, mortality, and overall. It also increases the chances of developing dementia. So at least we need to identify those patients who are frail, so that we can take some proactive measures.”* [F025, Anesthesia doctor]  *“If let’s say physically the patient is frail, then you will be very concerned because it’s the current– very hot topic in the hospital, the high fall risk. We seem like have almost– every day we have one fall risk incident happen in our hospital, and in a month we can have up to more than 30 cases, so it’s very alarming. This is in terms of physical frailty.”* [F046, Orthopedic nurse]  *“I think, frailty scores allow us to identify the frail patients and we know that this group of frail patients may not do as well as we like and perhaps, we can increase the level of service provided to them, during different phases of their treatment and recovery. There is some evidence that perhaps by, through identification of high-risk cases and increasing levels of services, you see, may potentially lead to a better outcome.”* [F018, General surgery doctor] |
|  | Patients with frailty requires modified treatment and/or more intensive clinical care to achieve better outcomes | *“This screening result is even helpful in operation preparation. Like let’s say if the surgery is not emergency, then, you know the patient is frail, I think you may want to start doing pre-rehabilitation which is rehabilitation before an operation. Published data shows frail patients should strengthen up their muscle mass before going for the surgery. It’s beneficial for frail patients.”* [F063, Orthopedic doctor]  *“It does impact me, at least in my practice, if I know the patient is frail. Because frail patients lose a lot more heat under anesthesia and the moment they go cold it’s very hard to warm them up again. So when we are looking after frail patients, we have increased– at least in my mind, we have increased our standard of care for our patients. Particularly things like keeping them warm, which is one of the factors that affect outcome.”* [F058, Anesthesia doctor]  *“I think frailty screening will be quite useful for us to plan more targeted and customized programs for rehabilitation and reconstruction if we knew the frailty in them.”* [F022, General surgery doctor]  *“I guess it is helpful. Then you will know which patients need the extra attention, yeah, and what precautions you might want to enforce.”* [F047, A&E nurse] |
|  | Frailty screening provides information for decision making and prognosis estimation | *“It provides at least a baseline for us to base our clinical decisions on, and then also it helps to determine how fast or how slow we can get the patient up and running, and you know, return to community.”* [F024 Orthopedic doctor]  *“It’s important for us to make our surgical decision. because as the patient score indicates less frail, we’ll be more inclined to do surgery for him because we want to get him back to his normal. For a CFS scored 8 or 9, maybe I don’t want to do surgery at all for him, you know. Maybe he’s not going to benefit from any kind of surgery. So it will affect my clinical decision.”* [F010, General surgery doctor]  *“Those that are frail actually have a steeper functional decline after surgery over the years. And also they have a higher chance of not being able to be discharged back home. They have to be discharged into a facility but not directly into home.”* [F003, General surgery doctor]  *“I think generally, not just our A&E but most emergency departments are also placing an increase emphasis on screening for these possibly frail patients and also to guide them in correct dispositions when they get admitted to the ward as well.”* [F006, A&E doctor] |
| Barriers and facilitators to frailty screening | Cooperation from patient/caregivers | *“You know patients come for the purpose of surgery and perhaps majority of them never heard about the concept of frailty. I guess they would not be willing to spend time to do the screening. They just want us to treat the diseases and get rid of the pain. So how come they would cooperate and do a screening they never knew.”* [F027, Orthopedic doctor]  *“But most of the time either the patients are in denial or they have a poor memory. We can’t get the proper answer from them, yeah. So that’s why we actually depend a lot on the caregiver. But it is also the challenge. Sometimes they dump and they leave.”* [F030, A&E doctor]  *“Both emotionally to give the answer to how much is their household income, versus do they really want to reveal this kind of information that indirectly would have implications on their frailty, but can still-they might not be comfortable in giving you this kind of information (in Tilburg Frailty Indicator).”* [F042, Anesthesia doctor]  *“Cause patient report, sometimes may not be accurate also. The family, sometimes report from them also may not be accurate.”* [F005, General surgery doctor] |
|  | Acceptance from healthcare workers/hospital managers | *“It’s not necessary to add this frailty screening. We do what we call system screening, so basically is going through the patient's medical history which I think is already forming a part of the frailty screening.”* [F021, Anesthesia doctor]  *“I mean personally it does not affect my decisions in terms of actual medical management for the patient. It might to a certain extent affect in terms of what is the extent of care, but the initial management is still the same.”* [F026, A&E doctor]  *“Definitely there’s a challenge where we have to get buy-in from everyone, from the doctors and nurses, to bring forward the CFS scoring that is to be done at triage.”* [F007, A&E doctor] |
|  | Dedicated resources | *“But you want to do a proper comprehensive geriatric screening like in the (hospital name omitted) A&E, (it) takes a long time and you need trained nurses. So it’s good to have, but not possible.”* [F030, A&E doctor]  *“Somebody will have to somewhere find the money and resources. It’s easy for me to say it should be done by geriatrician, but the geriatrician will say hey, have I got only your thing to do? I’ve got 1000 more other things to do.”* [F025, Anesthesia doctor]  *“This (frailty screening) is a challenge. I mean it really depends on who is the one that's doing it. Because currently, I mean there is no extra headcount to do this screening and every nurse is very busy already. Let's say you are the primary nurse, you one person has to take care of 12 patients, you have quite a lot work already.”* [F050, General surgery nurse] |
|  | Guidelines for frailty management | *“We screen, we pick up issues and there’s no intervention in the end. So I think it must be a smooth flow where everyone sits together and come up with the workflow.”* [F007, A&E doctor]  *“Yes, I have to admit that frailty screening is important. However, I haven’t heard about any anesthesia guideline on frail patients. I’ve no idea on what the next step is even though we have those frail patients identified.”* [F028, Anesthesia doctor]  *“As you noticed, we have the care protocol for fall prevention in our department. I think it will be a good idea if we have another protocol for frail inpatients as well.”* [F034, Orthopedic nurse] |
|  | Uniform scope of measurement among specialties | *“If the patient lands up with a surgeon, it is related to some surgical issue or something which is required, then we want a very objective scoring system which is not relying only on patient’s questions. So then the frailty index will be, for surgeons the best way to get a very objective scoring.”* [F040, General surgery doctor]  *“For us it’s a huge part to do with mental well-being. There’s some patients who– we done surgery and everything, physical we think they can manage but mentally they’re not fit enough.”* [F065, Orthopedic doctor]  *“So the frailty screening must be quite specific, in the sense that it’s targeted, and for me as a hand surgeon is to be able to have a summary of the patient’s cognitive function, and also the musculoskeletal and neuromuscular function, then I can plan something accordingly.”* [F004, Orthopedic doctor]  *“I agree that we should try to simplify as much as possible, but we also recognize that certain scales are not meant, designed for certain conditions right, so I can’t use Tilburg in the A&E, this will not work. I think the psychosocial is important but probably A&E is not the appropriate setting also to get that because sometimes in this kind of busy, crowded environment, also if you were to ask the patient like, ‘Do you often feel sad or depressed?”* [F001, A&E doctor] |
